# Impacts of early marriage and adolescent pregnancy on mental and somatic health: the role of partner violence

**DOI:** 10.1007/s00737-019-00960-w

**Published:** 2019-04-06

**Authors:** Aysen Ufuk Sezgin, Raija-Leena Punamäki

**Affiliations:** 1 Istanbul University, Istanbul Medical Faculty, Forensic Medicine Department, Istanbul, Turkey; 2 Faculty of Social Sciences/Psychology, Tampere University, Kalevankatu 5, Linna 4krs, FIM-33014 Tampere, Finland

**Keywords:** Early marriage, Adolescent pregnancy, Mental health problems, Somatic illness, Intimate partner violence

## Abstract

Researchers agree that early marriage (EM) and adolescent pregnancy (AP) can form severe risks for women’s somatic, mental, and reproductive health, as well as on educational and social status. Yet, less is known about factors that may moderate or mediate these associations. This study examined, first, retrospectively the impacts of EM and AP on self-reported mental and somatic health among multicultural group of women living in Eastern Anatolia, Turkey. Second task was to analyze whether and how the partner violence would mediate and/or moderate between EM and AP and mental health problems. The participants were 1569 women (16–72 years of age), who reported their age of being married, first pregnancy, and demographic characteristics. They described their mental health status through General Health Questionnaire (GHQ-28: depressive, anxiety, social dysfunction, and somatization symptoms) and symptoms of posttraumatic stress disorder (PTSD; DSM-5). Women’s reports of somatic illnesses were classified according to WHO-ICD-10. The revised conflict tactics scale, short form was used as a proxy to partner violence. Women who gave birth at 13–19 years of age reported more anxiety and somatization symptoms than later delivered, and those married younger than 25 showed a higher level of depressive symptoms than later married. Both AP and EM formed a heightened risk for somatic illnesses. The partner violence functioned as a moderator; AP was associated with especially high levels of depressive and anxiety symptoms among women exposed to sexual coercion in their marriage. Non-significant mediation analysis indicates that partner violence did not explain the severe impacts of the AP and EM on women’s mental health. Yet, the AP and EM were associated with heightened level of partner violence. Adolescent pregnancy forms a comprehensive mental health risk, and both AP and EM were risks for somatic illnesses, such as cardiovascular problems. The mental health risk of AP further intensified if women experienced sexual coercion in their partnership. Our fundamental work is to abolish these patriarchal phenomena.

## Introduction

Adolescent pregnancy (AP) and early marriage (EM) are considered public health and social risks, as well as individual tragedy. They are associated with mental health (Buzi et al. 2015), parenting (Secco and Moffatt 2003), and educational (Erulkar 2013) problems, as well as with obstetric risks (Azevedo et al. 2015). AP and EM reflect violation of human and women rights, and global injustice, as 90% of them happen in low- and middle-income countries (WHO 2011). Globally, over 700 million girls are married before 18 years of age, and child brides are common in low-income countries (UNICEF 2014).

The participants of the present representative study live in Eastern Anatolia of Turkey that is a poor, earthquake-affected, and politically and militarily unstable area. Official statistics of the prevalence of EM and AP are not available, but births to adolescents aged 10–19 years may be estimated to reach about 10%, thus similar to other low-income patriarchal societies (Ganchimeg et al. 2014; Kaplanoglu et al. 2015). It is sometimes argued that in patriarchal and traditional societies, EM is valued and normative, and AP symbolizes fertility and blessing, and therefore they would not be harmful to women (Harris-Short 2003; Locoh 1999). Characteristics to these societies are men’s privileges and their visible and subliminal dominance over women, manifested in the values, attitudes, customs, and expectations embedded in social institutions, and maintained through the socialization (Haj-Yahia 2003; International Encyclopedia of the Social Sciences 2018). The present study aims to learn about influences of EM and AP on women’s mental and somatic health, and about the role of violent partner relations in the context of a patriarchal and traditional society.

Augmenting evidence shows that AP increases obstetric and perinatal risks, such as low birth weight or prematurity (Bilano et al. 2014; Ganchimeg et al. 2013, 2014: Malabarey et al. 2012). A three-continent register study (23 low- and middle-income countries) found higher risks for low birth weight, preterm delivery, and severe neonatal conditions among 10–19 year-old mothers, as compared with older ones (Bilano et al. 2014; Ganchimeg et al. 2013, 2014). A population-based register study in the USA (37 million births) confirmed that very low and low birth weight, smallness for gestational age, fetal growth restriction, stillbirth, and infant death were significantly higher among mothers under 15 years (Malabarey et al. 2012). Similarly, a register study (*n* = 233) among Eastern Anatolian women evidenced that AP was an independent risk for heightened obstetric and perinatal problems, especially when the mother was under 15 years (Kaplanoglu et al. 2015). The maternal physiological immaturity formed a 2–3 times higher risk for low birth weight and fetal growth restriction, prematurity, and poor newborn health. The AP can also risk maternal health, and a US register study on pregnancies under 25 years (*N* = 43,537) found that that anemia and medical risks (e.g., hemorrhage, blood transfusion) were significantly higher in mothers under 16 years (Kawakita et al. 2016).

Research on impacts of AP on mental health is predominantly available from African American mothers, confirming especially high depression in pregnancy and postpartum, reaching to 40–50% of moderate-to-severe symptoms (Brown et al. 2012; Buzi et al. 2015; Meltzer-Brody et al. 2013). Similarly, AP is associated with high rates of depressive symptoms in European countries (Figueiredo et al. 2007; Reis and Källén 2010). Depression is shown to continue from the early years of AP parenting, and a 17-year-long follow-up found substantial increase in the severe depression among women with AP (Gavin et al. 2011). Concerning EM, it is agreed that the very early marriage age, often immediately after menarche, is causing also the highest mental health problems (UNPF 2013). There is evidence of suicide and self-harm among child brides worldwide (Gage 2013; Raj et al. 2008; Rasool and Payton 2014).

Research shows that women with EM and AP are highly vulnerable to domestic and partner violence (Chandra-Mouli et al. 2015), and some studies confirm that in low- and middle-income countries. An epidemiological study among Ethiopian women (*N* = 1671) found that the very early married (younger than 16 years) reported more partner assaults, sexual coercion, and communication problems than later married (Erulkar 2013). Our study analyses the mediating and moderating roles of partner violence in the association between EM and AP on women’s mental and somatic health. It has been estimated that globally about a third of women experience violence with their partners involving physical and psychological violence, and sexual aggression.

Domestic and partner violence are found to increase women’s mental health problems such as depression or even suicide, and to deteriorate somatic health (Bacchus et al. 2018; Devries et al. 2011), as well as to form a risk for physiological risk factors, such as high cardiovascular and stress reactivity (Robles et al. 2014). Concerning reproductive health, violence-exposed women are 16% more likely to have a low-birthweight baby, and twice as likely to have an abortion, as compared with women who have not experienced partner violence (Ellsberg et al. 2008: Chandra-Mouli et al. 2015; WHO 2013).). Yet, a prospective population-based study in South Africa found a bidirectional association, i.e., not only partner violence was associated with increased risk of future depression, but also depression was associated with increased risk of future violence in transition to motherhood (Tsai et al. 2016). We could detect few studies on how partner violence would impact on the mental health risks found among women with AP histories. African American adolescent mothers were at increased risk for severe depression, if they experienced partner violence, family conflicts, and lack of social support (Buzi et al. 2015; Reid and Meadows-Oliver 2007).

### Aims of the study

The first aim was to examine whether and how EM and AP are associated with women’s current mental and somatic health problems. We hypothesized that women married and giving birth at young age (10–19 years), and especially under 16, would report more PTSD, depression, anxiety and somatization symptoms, and social dysfunction, as well as more somatic illnesses, such as cardiovascular or muscular problems than women with later EM and AP. The second aim was to examine the mediating role of partner violence between EM and AP and mental health problems. According to the mediating hypothesis, the EM and AP would be associated with a high level of partner violence, which in turn would be associated with high levels of mental health problems. Third aim was to test the moderating hypothesis that EM and AP would form an especially high risk for mental health problems, if women experience a high level of partner violence.

## Method

### Participants and study procedure

The participants were 1569 women (16–72 years, mean 36.82 + 10.13) living in Eastern Anatolia. The sample represents 23 provinces of the region, selected 140 towns and districts according to Nomenclature of Territorial Units for Statistics (NUT1), and stratified according to the total population of each town/district. The ethical quality of the study plan and method was reviewed by the Women’s Centre in Diyarbakır (KA-MER 2004), an NGO (non-government organization) that provides multidisciplinary services for women and their families.

Forty female fieldworkers in each selected place entered every third house and asked one woman to join. They informed her about her rights, purpose of the research, and voluntary nature of the participation. If willing, the woman signed an informed consent or provided an oral approval in case she felt hesitant to give a signature. Except illiterate women, the participants were asked to fill in an anonymous self-administered questionnaire. The visits lasted 1–2 h, and the data collection was conducted in a separate room to provide privacy. The fieldworkers had extensive experiences in working with women and were familiar with the local cultures and languages. The first author and KA-MER professionals trained them on the research procedure, interviewing skills, and ethical rules, and arranged bi-weekly supervising meetings. The aims were to ensure consistency in the data collection procedures and to enhance fieldworkers’ well-being and resources.

### Measures

#### Background information

Women reported their age, education, civil status, economic stand, work occupation, number of children, and ethnic background.

#### Early marriage (EM) and adolescent pregnancy (AP)

Women reported to an open question at what age they were married and what age they gave birth to their first child. The ages were categorized according to international standards.

#### Posttraumatic stress disorder symptoms

The nine-item National Stressful Events Survey PTSD Short Scale (NSESSS-PTSD) (LeBeau et al. 2014) is based on the DSM-5 diagnostic criteria and covers the dimensions of intrusion, avoidance, and hypervigilance, as well as negative cognitive-affective responses. Women answered on a 5-point scale to what extent they had the symptoms during the previous week (0 = *not at all*; 4 *= extremely*). A total sum score was constructed and the internal consistence by Cronbach’s *α*-value was .89. A clinical cut-off score was also calculated based on the score 24 (LeBeau et al. 2014). A student-based study found the Turkish version of the NSESSS-PTSD to be psychometrically sound PTSD screening measure with high convergent and discriminant validity, and reliability as well as good sensitivity and specificity (Evren et al. 2016).

#### Psychiatric distress

General Health Questionnaire (GHQ-28) is built to assess psychiatric disorders in the general population (Goldberg et al. 1997), including Turkey (Kılıç et al. 1997). Women estimated how well the 28 descriptions fit their feelings, behavior, and thinking during the last week (1 = *not at all*; 4 = *much more than usual*). Total sum scores were created for depressive (7 items; *α* = .90), anxiety (7 items; *α* = .91), and somatization (7 items; *α* = .91) symptoms, and social dysfunction (7 items; *α* = .88). The clinical cut-of 5 (after dichotomizing) was calculated according to Kadıoğlu et al. (2013).

#### Somatic illnesses

Women reported about their illnesses to an open question. The responses were classified according to WHO-ICD-10 diseases and disorders classification. The women also reported their usage of regular medication, but their purpose was not inquired.

#### Partner violence

The revised conflict tactics scale, short form (CTS2S; Straus and Douglas 2004) was used to assess the frequency and severity of physical assault, injury, sexual abuse, and psychological aggression in the marital relationship. The 10-item scale covers dimensions of physical assaults (e.g., causing bruises and wounding), sexual coercion (e.g., rape with physical force), psychological aggression (e.g., insulting and yelling), and negotiation (reversed, e.g., respecting for or caring about feelings). Women reported how often the described behaviors occurred separately for the spouse and themselves: 0 = never happened, 1 = not in the last year, but it did happen before, 2 = once in the past year, 3 = two times in the past year, 4 = 3–5 times in the past year, 5 = 6–10 times in the past year, 6 = 11–20 times in the past year, and 7 = more than 20 times in the past year. The dimensionality of the CTS2S was checked by principal component analysis (varimax rotation with Kaiser normalization). Results revealed three factors instead of the original four, as psychological aggression items loaded on physical assaults. Six total sum scores were constructed separately for partner violence by the spouse and by woman herself and named for both as (1) physical and psychological assault, (2) sexual coercion, and (3) uncaring (negotiation scale reversed). These three factors explained 45.7% of the total variance.

#### Translation

The GHQ-28 and conflict tactics scale (original with the same items) were available in Turkish. The NSESSS-PTSD-scale was translated from English to Turkish, and back translated by a bilingual psychologist. All scales were piloted in the KA-MER.

### Statistical analysis

Multivariate analysis with covariance (MANCOVA), followed by univariate analyses of ANCOVA and Tukey-b post hoc tests, was used to compare the levels of PTSD, depression, anxiety and somatization symptoms, and social dysfunction between very early (10–15 years), early (16–19 years), and later (20–25 years and > 25 years) married or delivering women (EM and AP). Woman’s age, education (dummy variable, 0 = no education; 1 = education) and economic status (0 = no income; 1 = income) were the covariates as AP and EM differed according to them (analyses available from the authors). Crosstabs with *χ*^2^ statistics tested whether EM and AP were associated with somatic illnesses (dichotomy variables of the ICD-10 diagnoses).

The Structural equation modeling (SEM; AMOS 15.0 software; SPSS Framework Version) was used to analyze the mediating role of the partner violence (CTS2S-scale as a proxy) between EM and AP, and mental health. AMOS uses a maximum likelihood method for obtaining estimates of the parameters for both direct and indirect/mediated paths. As exogenous latent construct of EM and AP (manifest variables of dummy EM and dummy AP; 0 = > 16 years, 1 = < 16 years) was regressed to the latent construct of women’s mental health (manifest variables: PTSD, depression, anxiety, and somatization symptoms, and social dysfunction), two endogenous latent constructs, partner violence by the spouse and by the woman herself (both with three manifest variables of physical and psychological assaults, sexual coercion, and uncaring), were entered as possible mediators. The measurement models of the latent constructs were first tested to confirm that the manifest variables loaded significantly. The criteria for model fitness were non-significant *χ*^2^ value, comparative fit index (CFI) and normative fit index (NFI) > .90, and RMSEA < .06 (Bentler 2007). The residuals of the two endogenous latent constructs of the partner violence by the spouse and by the women herself were allowed to covary to reflect shared sources of variance not included in the model.

To test the moderating role of the partner violence (combined sum variables of violence by the spouse and the woman herself), stepwise regression analyses were used. At first step, woman’s age, dummy variables of education, and economic stand were entered as control variables, then at the 2nd step, the main effects of EM and AP dummy variables, and at the 3rd step, the three sum variables of the partner violence were entered. At 4th step, the interaction terms of EM * IPV and AP * IPV were entered. The interaction variables were first centered to avoid multicollinearity (Aiken et al. 1991). The PTSD, depressive, anxiety, and somatization symptoms, and social dysfunction were the dependent variables. MANCOVA, *χ*^2^ statistics, and regression analyses were done by SPSS 23.0 for Windows.

## Results

### Descriptive statistics

Table 1 shows that about a half (49%) of women were 36–59 years old, and a third were 25–35 years. Less than a tenth were unmarried (9%; *N* = 143). EM and AP were common, as 47% were married at the age of 16–19 and 14% at the age of 10–15 years; 4% had given birth when 15 or younger, and 41% before their 20th birthday. The average child number was 3.45 (SD = 1.88) and 61% of women had 3–7 children. About a third (32%) did not have a formal education, and 6% had a university degree. The families were poor, about a half (54%) earned less than 1000 Turkish lira (280 USD) a month, and 10% of women reported not having any (own) regular income. The sample involved five ethnic groups, the largest was Kurdish (52%), a third were Turkish, and the rest Zazas, Arabs, and others.

**Table 1:** Percentages and frequencies of demographic characteristics

	Women sample	
	%	*n*
Age		
15–18	1.4	17
19–24	10.3	161
25–35	36.6	571
36–59	49.2	768
60–72	2.8	43
Marriage age^a^		
10–15	14.4	206
16–19	46.8	667
20–25	31.3	446
Older than 26	7.5	107
Not married	9.1	143
First child birth^a^		
13–15	4.1	52
16–19	41.3	525
20–25	42.8	545
Older than 25	11.8	150
Number of children^a^		
None	0.6	8
1–2	34.2	460
3–7	61.2	822
8 or more	4.0	54
Education		
No formal schooling	31.8	494
Primary school	47.0	730
High school	13.7	213
University degree	5.6	87
Other	1.8	28
	%	N
Family monthly income^b^		
Very low	14.4	221
Low	39.3	603
Moderate	25.6	393
High e	9.5	145
No income	10.0	153
Does not know	1.2	18
Source of income		
No own income	69.4	1048
Works in paid job	12.3	186
Other income	17.7	276
Working situation		
Unemployed	16.1	243
House wife or student	73.2	1105
In working life	10.7	161
Working status of spouse		
Not working	27.0	348
Half time	12.6	162
Full time	59.1	762
Student	0.2	3
Retired	1.1	14
Ethnic origin		
Turkish	36.4	567
Kurdish	51.6	803
Zaza	6.0	94
Arabic	4.4	69
Other	1.5	24

Differences in numbers are due to missing values

^a^The distribution concerns women who are married (91.1%; *N* = 1418)

^b^Based on monthly income in Turkish lira (TRY): very low (< 500TL); low (500–999TL); moderate (1000–2499); high (2500TL or more); 1 USD = 3.80 TRY

There was naturally a significant association between EM and AP (*χ*^2^ (9,1271) = 1479.13, *p* < .0001), indicating that women often gave birth soon after marriage. For example, of women married at 16–19 years of age, 65.5% gave birth during the same age period, and among those who married at 20–25 years of age, the share was 83.4%. Yet, women who married at 10–15 years of age, 68% gave birth at 16–19 years.

Of women, 27.3% (*n* = 426) reported some somatic illness. Table 2 specifies that musculoskeletal system disorders, such as back pain, were the most common (32%) and almost a fifth (18%) had hematological system diseases, mostly anemia. A fifth of the women reported using regular medication (21.4%, *n* = 332). Of women, 27.6% showed clinically significant PTSD symptoms, and 27.2% scored above the clinical cut-off point in GHQ-28.

**Table 2:** Percentages and frequencies of somatic illnesses

	Women’s own reporting	
	%	*n*
Circulatory system diseases	14.9	62
Respiratory system diseases	12.9	54
Musculoskeletal system disorders	32.3	134
Hematological diseases	17.6	73
Mental and neurological disorders	10.8	45
Other illnesses	10.5	44

Women could report more than one disease or disorder. Totally, 27.3% (*n* = 426) of women reported somatic illnesses, but not all specified them

About a third of women reported physical and psychological assaults and sexual coercion by their spouses. For instance, 36.5% had experienced kicking and beating, 36.1% pushing and slapping, and 30.2% threats to rape sometimes and often. The corresponding occurrences of these violent acts by IPV woman herself were 6%, 9.5%, and 2.7% respectively.

### Early marriage, adolescent pregnancy, and mental and somatic health

MANCOVAs showed that mental health problems differed according to both AP (*F*_Roy’s Largerst Root_ (51242) = 4.97, *p* < .0001, *η*^2^ = .02) and EM (*F*_Roy’s Largerst Root_ (51242) = 2.49, *p* < .03, *η*^2^ = .03). Results in Table 3 specify, however, that the AP formed a more comprehensive risk for women’s mental health than the EM. Both women who gave birth very early (13–15 years) and early (16–19 years) showed higher levels of anxiety (*F*(3,1244) = 3.26, *p* < .021, *η*^2^ = .01) and somatization (*F*(3,1244) = 4.78, *p* < .003, *η*^2^ = .01) symptoms than women who delivered in older ages. However, women who gave birth to the first child in both very early/early ages and later than 25 years showed higher levels of depressive symptoms than those giving birth at 20–25 (*F*(3,1244) = 6.17, *p* < .0001, *η*^2^ = .02), indicating curvilinearity. Only depressive symptoms differed according to EM, showing, interestingly, that women married younger than 26 years reported significantly higher levels of depressive symptoms than later married (*F*(3,1244) = 3.42, *p* < .02, *η*^2^ = .01).

**Table 3:** Age of marriage and age of first child birth and mental health problems: adjusted means, standard errors, and ANCOVA and post hoc statistics^a^

	PTSD symptoms		Depressive symptoms		Anxiety symptoms		Social dysfunction		Somatization symptoms	
	M	SE	M	SE	M	SE	M	SE	M	SE
Age of marriage										
10–15 years	2.18	.09	2.36^a^	.07	1.60	.06	2.09	.05	2.36	.07
16–19 years	2.02	.07	2.23^a^	.05	1.59	.05	2.04	.04	2.25	.05
20–25 years	2.02	.08	2.26^a^	.06	1.63	.05	2.03	.05	2.33	.06
> 25 years	1.84	.16	1.91^b^	.13	1.48	.11	1.90	.09	2.19	.13
*F*-value (31244)	1.31		3.42*		0.68		0.87		1.49	
Age of first child birth										
13–15 years	2.03	.17	2.27^a^	.13	1.75^a^	.12	2.16	.01	2.43^a^	.13
16–19 years	2.08	.07	2.28^a^	.06	1.64^ab^	.05	2.02	.04	2.39^a^	.06
20–25 years	1.91	.06	2.04^b^	.05	1.47^bc^	.05	2.00	.04	2.14^b^	.05
> 25 years	2.03	.10	2.27^a^	.08	1.43^c^	.07	1.98	.06	2.16^b^	.08
*F*-value (31244)	1.56		6.17***		3.26*		0.12		4.78*	

*p < .05, ***p < .001

^a^Tukey-b post hoc statistics should be read column-wise, the different upper letters indicate statistically significant (*p* < .05) differences between the mean values. The analyses were covariated for age, education, and economic stand

Both EM and AP differed in the occurrence of somatic illness (*χ*^2^ (3,1421) = 15.16, *p* < .002 and *χ*^2^ (3,1421) = 17.41, *p* < .001, respectively). More than a third (39.2%) of women who had married at very early age (10–15) reported some diagnosed somatic illness, while the share was 25.2% among later married. Also, more than a third of women delivering at the very early age (39.2%) and early age (35.0%) reported somatic illnesses, while the occurrence was 25.8% among later delivered. Regular medication was also more common among EM (*χ*^2^ (3,1415) = 16.68, *p* < .001) and AP (*χ*^2^ (3,126) = 12.72, *p* < .005) women than later married and delivered.

### Role of the partner violence

**Fig. 1:**
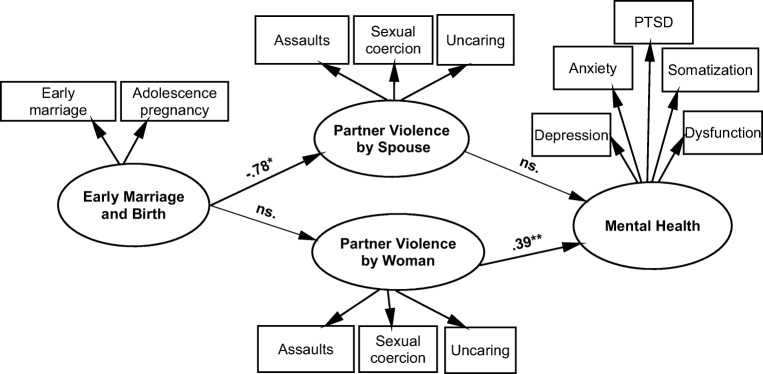
Structural equation model (SEM) results of early marriage (EM) and adolescent pregnancy (AP) on women’s mental health problems mediated by the intimate partner violence (separately for spouse and woman herself). Non-significant paths are shown with dashed lines, and error terms and correlated errors are not shown, as they are available in Table 4

Concerning the mediating hypothesis, the measurement models, direct and indirect SEM paths, and fit indices are presented in Table 4. The model fits the data well (CFI and NFI > .90, RMSEA < .06), although *χ*^2^ was significant, which is common in large samples (Bentler 2007). The results rejected the hypothesis that EM and AP would be associated with women’s mental health problems via high level of partner violence. Figure 1 shows that although the latent construct of EM and AP was significantly associated with a high level of the partner violence by spouse (*β* = − .78, CR = 2.30, *p* < .05), that violence did not further associate with women’s mental health problems. The violence by woman herself in turn was significantly associated with her mental health problems (*β* = .39, CR = 3.27, *p* < .01), but the path between the EM/AP construct and that violence was non-significant.

**Table 4:** Structural equation model (SEM) on mediation of intimate partner violence between early marriage (EM) and adolescent pregnancy (AP) on women’s mental health: parameter estimates (coefficients) associations and model fit indices

	Total sample (*N* = 1569)			
	Unstandardized estimate	Standardized estimate	S.E.	Critical ratio
Measurement model of early marriage and birth^a^				
Early marriage - > early marriage and birth		.71		1.00
Age of first child birth - > early marriage and birth	0.73	.55	0.43	16.75****
Measurement model of mental health				
PTSD symptoms - > mental health		.59		1.00
Depressive symptoms - > mental health	1.10	.82	.05	22.91****
Anxiety symptoms - > mental health	0.88	.73	.04	21.34****
Somatization symptoms - > mental health	1.23	.91	.05	23.96****
Psychosocial functioning - > mental health	0.64	.64	.03	19.45****
Measurement model of intimate partner violence: spouse behavior				
Physical and psychological assault	2.41	.19	.65	3.73****
Sexual coercion	0.43	.15	.03	1.56
Uncaring		.08		1.00
Measurement model of intimate partner violence: woman behavior				
Physical and psychological assault	3.82	.55	1.73	2.21*
Sexual coercion		.14		1.00
Uncaring	0.37	.09	.27	1.36
Structural equation model: direct path				
Early marriage and birth - > mental health	0.45	.05	1.62	0.28
Structural equation model: indirect paths				
Early marriage and birth - > partner violence: spouse behavior	− 0.02	− .78	.009	− 2.30*
Early marriage and birth - > partner violence: woman behavior	− 0.01	.12	.01	− 1.37
Partner violence: spouse behavior - > mental health	2.96	.10	.25	0.29
Partner violence: woman behavior - > mental health	1.70	.39	.52	3.27**
Model fit indices	*χ*^2^ (67) = 397.22 *p* = .0001			
	NFI = 94, TLI = .93, CFI = .95			
	RMSEA = .059 (90% CI .053–.065)			

*p < .05, **p < .01, ****p < .0001^a^

**Fig. 2:**
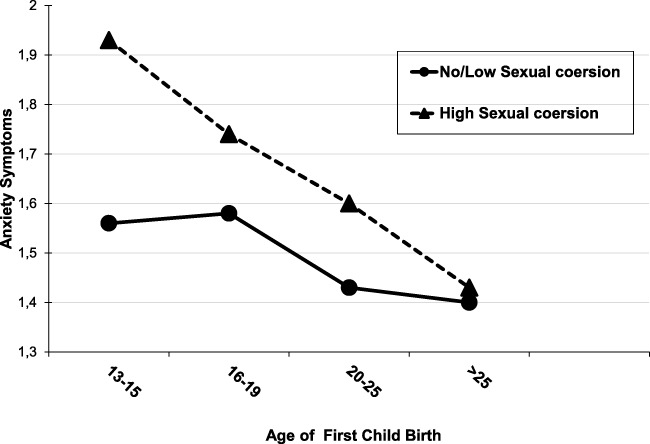
The association between adolescence pregnancy (AP) and anxiety symptoms according to sexual coercion as partner violence

Instead, the moderation hypothesis was confirmed for sexual coercion and AP. Regression analyses in Table 5 reveal significant interaction effect between the AP and partner violence on depressive and anxiety symptoms, as indicated by the statistically significant fifth step (change in *F*-values) and *β*-values. The total regression model explained 15–18% of the variation of these symptoms, and the interactions between AP and Sexual coercion were significant on depressive (*β* = − .15, *t* = − 2.50, *p* < .01) and anxiety (*β* = − .17, *t* = − 2.72, *p* < .007) symptoms. As hypothesized, AP formed an especially high risk for these mental health problems, if women experienced sexual coercion in their marriage. Figure 2 illustrates schematically that AP (giving birth at 13–15 years) was associated with higher levels of anxiety symptoms among women who reported a high level of sexual coercion. The hypotheses were rejected concerning the role of partner violence in the association between EM and mental health.

**Table 5:** Main and interaction effects of partner violence on women’s mental health (General Health Questionnaire (GHQ-28))

	Depressive symptoms					Anxiety symptoms				
	*R*^2^	*F*-valueΔ *R*^2^	*Β*	StdE	*β*^d^	*R*^2^	*F*-value Δ *R*^2^	*Β*	StdE	*β*^d^
I Control variables	.06	24.88****				.02	8.08****			
Woman’s age			0.01	.00	.07*			− .00	.00	− .03
Education^a^			− 0.22	.05	− .13****			− .09	.04	− .06*
Economic stand^a^			− .38	.07	− .15****			− .24	.06	− .11****
II Women’s risks	.08	7.63***				.03	6.71***			
Early marriage EM^b^			− .04	.07	− .03			− .02	.06	− .01
Adolescent pregnancy AP^b^			.19	.06	.12***			.14	.06	.10**
III Intimate partner violence^c^	.12	18.08****				.08	19.89****			
Physical and psychological assault			.17	.02	.21****			.15	.02	.21****
Sexual coercion			.06	.02	.08**			.05	.02	.07*
Uncaring			.02	.02	.02			.06	.02	.08***
IV Interaction effects for EM	.12	0.32				.09	0.51			
EM* physical, psychological assault			− .09	.07	− .05			− .09	.06	− .07
EM* sexual coercion			− .20	.09	− .14*			− .18	.08	− .14*
EM* uncaring			− .04	.07	− .03			− .03	.06	− .02
V Interaction effects for PA	.13	2.84*				.10	3.55**			
PA* physical, psychological assault			− .07	.07	− .04			− .09	.06	− .06
PA* sexual coercion			− .24	.10	− .15**			− .24	.09	− .17***
PA* uncaring			− .05	.06	− .03			− .01	.06	− .01
Models	*F* (14,1086) = 11.38, *p* < .0001; 13% explained variance					*F* (14,1086) = 8.03, *p* < .0001; 10% explained variance				
	Social dysfunction					Somatic symptoms				
	*R*^2^	*F*-value Δ *R*^2^		StdE	*β*^d^	*R*^2^	*F*-value Δ *R*^2^		StdE	*β*^d^
I Control variables	.03	10.46****				.06	21.56****			
Woman’s age			.00	.00	.09***			.00	.00	.04
Education^a^			− .06	.04	− .04			− .18	.05	− .10***
Economic stand^a^			− .20	.05	− .11****			− .43	.07	− .17****
II Women risks	.03	1.40				.08	11.78****			
Early marriage EM^b^			.03	.05	.02			−.07	.07	−.04
Adolescent pregnancy AP^b^			.02	.05	.01			.26	.07	.16****
III Intimate partner violence^c^	.07	13.65****				.15	29.87****			
Physical and psychological assault			.11	.02	.18****			.22	.02	.26****
Sexual coercion			.01	.02	.02			.03	.02	.04
Uncaring			.03	.02	.05*			.02	.02	.02
IV Interaction effects for EM	.07	0.52				.15	0.40			
EM* physical, psychological assault			− .00	.05	− .00			− .11	.07	− .07
EM* sexual coercion			.09	.07	.09			− .04	.09	− .03
EM* uncaring			− .02	.05	− .01			.00	.07	.00
V Interaction effects for PA	.07	0.91				.15	1.09			
PA* physical, psychological assault			.05	.05	.04			− .12	.07	− .07
PA* sexual coercion			− .08	.07	− .07			− .01	.10	− .00
PA* uncaring			.04	.05	.03			− .02	.07	− .01
Models	*F* (14,1086) = 5.75, *p* < .0001; 7% explained variance					*F* (14,1086) = 13.61, *p* < .0001; 15% explained variance				

^a^Education (dummy variable 0 = no education; 1 = education) and economic status (dummy variable 0 = no income; 1 = income); ^a^EM and AP dummy variables (0 = older than 16 years, 1 = younger than 16 years); ^c^intimate partner violence by spouse; *β*-values are from the final fifth step of the regression models*p < .05, **p < .01; ***p < .001, ****p < .0001

Additionally, the significant main effects show that physical and psychological assaults were significantly associated with high levels of all mental health problems, and sexual coercion with high levels of depressive and anxiety symptoms. A high level of uncaring as a tactics to solve conflicts was associated with high levels of anxiety and social dysfunction.

## Discussion

Our findings confirmed that especially adolescent pregnancy (AP) formed a severe risk for women’s mental health, and both AP and early marriage (EM) for somatic health. Earlier research has focused predominantly on depression as mental health consequences of AP, but our results signify that the impacts can be more comprehensive. Women who gave birth before their 20th birthday showed higher levels of depressive, anxiety, and somatization symptoms than women delivering later. Women with both AP and EM suffered more of somatic illnesses and used regular medication. The experience of partner violence was confirmed to be a significant moderator, as AP was associated with high levels of depressive and anxiety symptoms especially among women who experienced sexual coercion in their marriage. Instead, the partner violence did not explain (mediate) the impacts of the AP and EM on women’s mental health problems, although AP and EM were associated with a higher level of partner violence by the spouse.

We expected that especially AP and EM in the very early age, under 16 years, are the most harmful for women’s mental health. Yet, it is noteworthy that also the early age (16–19 years) of AP formed a mental health risk, and marriage before 25 years was associated with high level of depressive symptoms. The findings contrast the research both in high- and low-income countries that emphasizes that especially the very early age of birth-giving and being a child bride form severe risks for women’s reproductive and somatic health (Kaplanoglu et al. 2015; Kawakita et al. 2016; Malabarey et al. 2012; Raj et al. 2008; Rasool and Payton 2014). Social and political aspects may help understand why AP at 16–19 years and marriage before 25 were mental health risks among our participating women. In patriarchal and hierarchic cultures of Eastern Anatolia, very few young girls can decide themselves about the timing of their marriage or child caring. Becoming a mother at 16–19 years of age means great doubts and insecurities, even if it is considered normative (Harris-Short 2003). Legal marriage age in Turkey is 18, and thus younger mothers are completely with mercy of their extended families. A sense of mastery, justice and meaningfulness can be regarded as universal ingredients of good mental health (Almedom 2005). However, women in Eastern Anatolia are commonly deprived from their very rights to decide about their bodies, minds, and future, as well as from their right to articulate their own preferences. An interview study found that protecting the family honor and economic considerations were the main reasons for EM and AP in Eastern Anatolia (Ertem and Kocturk 2008). Women revealed experiences of family tension and psychological strain due to parental arrangements of cousin and bride exchange marriages. A great part felt deeply wronged because of being extricated from the important decisions concerning their own lives.

Women who married and gave birth at very early age had especially poor somatic health and used regular medication. Of them, almost 40% suffered from various somatic illnesses such as hematological or circulatory system diseases, while a quarter reported these among later married and delivered women. The reasons for the somatic risk may partly relate to adolescents’ biological immaturity (Bilano et al. 2014; Kaplanoglu et al. 2015), stressful conditions, and age- and status-related inequalities between spouses (Brown et al. 2012; Buzi et al. 2015; Phipps et al. 2002).

Researchers argue that in traditional societies, people manifest their psychological pain preferably in somatic symptoms, because the cultural values enhance harmony and collective good, and because mental disorders are considered shameful and frightening (Kirmayer et al. 1993). Our findings specify, however, that somatization symptoms or somatic illnesses were not especially high among women living in traditional societies. They rather expressed a variety of symptoms, meaningfully comprising psychological, social, and somatic problems. The observation concurs with the critics that challenge the dichotomy between culturally collectivists and individualistic symptom manifestation (Brewer and Chen 2007).

We expected that EM and AP would be especially harmful for women’s mental health, if they are exposed to partner violence. Our results confirmed the hypothesis concerning the negative impact of AP, but not EM on women’s mental health and for sexual coercion, but not for physical and psychological assaults. Women who became mothers in very early or early age showed especially high levels of depressive and anxiety symptoms, if they suffered sexual coercion in their marriage. The significant main effects indicate that physical and psychological assaults and sexual coercion were also alone directly deteriorating women’s mental health.

The sexual coercion involves highly humiliating and frightening conducts in marriage. Sexuality is an intimate, personal, and vulnerable life domain, and sexual abuse, rape, and other sexually degrading experiences are severely traumatizing. Research confirms that interpersonal sexual trauma forms the highest risks for PTSD, depression, and symptom comorbidity (Kaltman et al. 2010; McMillan and Asmundson 2016). Women with AP and high partner violence were vulnerable to anxiety and depression. Anxiety symptoms encompassing physiological arousal, unhappiness and general apprehension, and depression with withdrawal and despair can severely interfere with women’s and their children’s quality of life. It is thus of utmost importance to abolish child marriages and pregnancies as well as violence against women, as a part of our struggle for human rights and security.

The finding of the intensifying mental health risks of sexual coercion such as rape in marriage is especially striking in the Turkish context, where in 2016, the government was pressured to withdraw a law bill that was drafted to pardon men who were convicted of having sex with underage girls if they would marry them. Although not legal, the patriarchal practices continue. If a family decides to marry a girl, she may be forced to engage in sexual intercourse, actually to rape, and physical violence (Jensen and Thornton 2003). The traditions of great gender inequality and suffering are thus present despite the fact that child marriage is itself a human right violation and criminal pedophilic act.

The hypothesis that partner violence would mediate between EM and AP and mental health problems was not confirmed, in other words, these human right violations were directly in themselves a risk for women’s well-being. We modeled separately the violence by the spouse and the woman herself, and interestingly, the results revealed that AP and EM were very strongly associated with the spousal violence (the *β*-value being as high as .78), which corresponds with earlier research showing the AP and EM as a risk for partner and domestic violence (Chandra-Mouli et al. 2015; Erulkar 2013). Reviews on the gender differences in partner violence suggest that men penetrate generally more violence in marriage, and women engage in psychological forms of partner violence (Chan 2011). This was also the case in our study, as about a third of married women reported husband-penetrated violence, while the share of women’s own violence was few percentages. Yet, the women-penetrated violence was significantly associated with their mental health in the mediating model including AP and EM. Our research setting is cross-sectional, and thus we cannot conclude whether women mental health problems resulted in violence or as hypothesize the vice versa. The South African prospective study across transition to motherhood showed reciprocal influences between partner violence and depression (Tsai et al. 2016).

The study deserves criticism for the single-informant self-reports and cross-sectional setting. Clinical interviews of women’s PTSD, depression, and anxiety would have provided more reliable information due to their more objective nature. A prospective study setting with the obstetric registry of the AP and epidemiological data of EM would provide a more comprehensive view for understanding the phenomena in these ethnic groups and cultures. Women reporting both their own and their spouse’s violent conduct may involve under- or/and overestimations that our statistics could not control. Furthermore, we did not have a valid measure to directly account the severity or frequencies of partner violence. We had to rely on the proxy of the phenomenon and ask women about the conflict solving strategies in their marital relationship. Direct asking would not have been respectful and safe in the context of traditional, hierarchical family relationships.

To conclude, adolescent pregnancy forms a severe mental health risk, and, together with early marriage, threatens women’s somatic health. Thus, both are considered a grave human right violation. The mental health risk is intensified when women experience sexual coercion. The fundamental work is to abolish these patriarchal phenomena. It is highly appreciated that voluntary NGOs offer professional help to the young women and girls, but help is also needed from the international forum, the society, the state, and the politicians to fight against the illegal practices.
